# A Disease‐Specific Simulator of the Laparoscopic Percutaneous Extraperitoneal Closure (LPEC) Method for Pediatric Inguinal Hernia: A Validation Study With Comparison of Experienced Pediatric Surgeons and Novices

**DOI:** 10.1002/wjs.70119

**Published:** 2025-10-05

**Authors:** Masakazu Murakami, Yumiko Tabata, Yumiko Iwamoto, Masato Ogata, Lynne Takada, Chihiro Kedoin, Yudai Tsuruno, Koshiro Sugita, Keisuke Yano, Shun Onishi, Takafumi Kawano, Satoshi Ieiri

**Affiliations:** ^1^ Department of Pediatric Surgery Research Field in Medical and Health Sciences Medical and Dental Area Research and Education Assembly Kagoshima University Kagoshima Japan; ^2^ Department of Pediatric Surgery Hokkaido Medical Center for Child Health and Rehabilitation Sapporo Japan

**Keywords:** disease‐specific simulator, inguinal hernia, LPEC, off‐the‐job training, pediatric endoscopic surgery, validation study

## Abstract

**Background:**

Laparoscopic inguinal hernia repair is the most common procedure in the pediatric field and is one of the first pediatric endoscopic surgical procedures that a trainee should learn. Off‐the‐job training is important in pediatric surgery to compensate for the lack of experience due to the small number of cases. We developed a disease‐specific simulator for laparoscopic percutaneous extraperitoneal closure (LPEC) of inguinal hernia. We compared the results of simulated surgery using this simulator between experts and novices.

**Methods:**

The simulator was developed for a 1‐year‐old infant body size and allowed for training in needle manipulation unique to LPEC. The participants were pediatric surgeons and trainees and were divided into novice and experienced groups. The task involved simulated LPEC operations. The task completion time, three‐dimensional characteristics of needle device manipulation evaluated by a magnetic three‐dimensional position‐measuring device, and accuracy of needle device manipulation evaluated by a checklist were compared. A questionnaire survey was conducted to evaluate the effectiveness of the simulator.

**Results:**

There were 35 and 18 participants in the experienced and novice groups, respectively. The experienced group had a significantly shorter task completion time than the novice group (308.1 vs. 695.8 s, *p* < 0.001), shorter total pass length (37591.1 vs. 102678.1 mm, *p* = 0.001), slower average velocity (79.2 vs. 136.8 mm/s, *p* = 0.002), and lower average acceleration than the novice group (8477.9 vs. 17775.5 mm/s^2^, *p* = 0.005). The experienced group showed significantly better performance in the evaluation using a checklist. In the questionnaire survey, the effectiveness of the simulator was highly evaluated by experienced surgeons.

**Conclusion:**

Our simulator could clearly differentiate between novice and experienced surgeons, and the validity of the LPEC simulator was established. The validity of the LPEC simulator was also proven using a questionnaire survey after simulation surgery.

## Graphical abstract

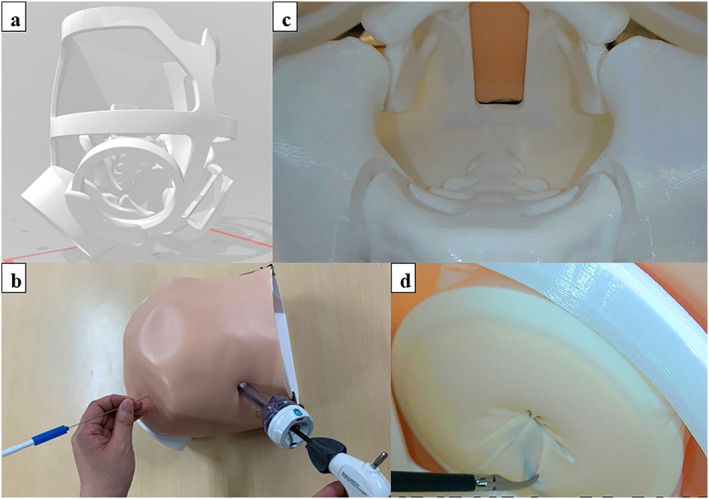

**Abbreviations**
LPEC: laparoscopic percutaneous extraperitoneal closure
SBT: simulation‐based training
SDs: standard deviations

## 1 Introduction

Endoscopic surgery has become widespread in the field of pediatric surgery. Laparoscopic inguinal hernia repair is the most common procedure in the pediatric field and the first pediatric endoscopic surgical procedure that a trainee should learn. Among laparoscopic surgeries for pediatric inguinal hernias, the laparoscopic percutaneous extraperitoneal closure (LPEC) method, developed in Japan, is not only cosmetically superior, but also a simple and secure procedure [1]. However, it requires special techniques, such as needle device manipulation through the abdominal wall under laparoscopic observation, and there is a serious risk of accidental injury to the external iliac artery and vein.

In comparison to general surgeons treating adult patients, pediatric surgeons have limited opportunities to perform endoscopic surgery because of the small number of cases. Off‐the‐job training is very important in pediatric surgery to compensate for the lack of experience due to the small number of cases, and in pediatric endoscopic surgery, simulation‐based training (SBT) is a highly effective educational modality [2]. However, effective off‐the‐job training models for LPEC have not yet been reported. Therefore, we developed a disease‐specific simulator for LPEC that compensates for the lack of experience and allows novices to learn secure needle‐device manipulation.

In the present study, to validate our disease‐specific simulator for LPEC, we compared the results of simulated surgery using this simulator between experts and novices, mainly with regard to needle manipulation.

## 2 Materials and Methods

### 2.1 Disease‐Specific Simulator for LPEC Surgery

**FIGURE 1:**
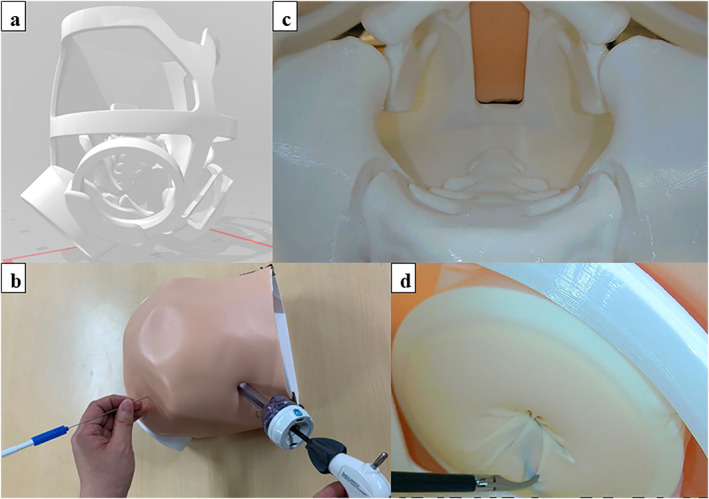
LPEC simulator. (a) 3D CAD data of the LPEC simulator. (b) The LPEC simulator with artificial skin. (c) The narrow pelvic cavity of the simulator. (d) Manipulation of the needle device under laparoscopic vision to pass the suture around the hernia sac.

We developed a disease‐specific simulator for LPEC surgery in collaboration with anatomical models, simulators, and imaging phantom companies (Kyoto Kagaku Co. Ltd., Kyoto, Japan). This simulator was developed with a 1‐year‐old infant body size based on computed tomography data and reproduced a pneumoperitoneum condition based on 3D scanning data of the body surface after the introduction of pneumoperitoneum (Figure 1a). The frame created using a 3D printer was covered with artificial skin made of silicone (Figure 1b). The infant's narrow pelvic cavity and lower abdomen were reproduced with pelvic bones created using a 3D printer (Figure 1c). The abdominal wall was reproduced using a sponge, and the peritoneum and hernia sac were reproduced using surgical gloves. The gonadal vessels and spermatic duct were reproduced with a silicon cord around the hernia sac (Figure 1d).

In the LPEC method for pediatric inguinal hernia, a needle device that can hold suture material at the tip of the needle with a wire loop is used for circuit suturing around the internal inguinal ring [1]. In our simulator, the trainees can pierce the needle device from the surface of the simulator and manipulate the needle device under laparoscopic vision to pass the suture around the hernial sac and ligate it extracorporeally (Figure 1d). For each training session, only the surgical glove needed to be replaced, allowing repeated training with inexpensive materials.

### 2.2 Study Participants

Participants were pediatric surgeons and trainees recruited at the conference venue of the 60th Annual Congress of the Japanese Society of Pediatric Surgeons in 2023. Participants were divided into two groups according to the number of experiences with LPEC surgery: the novice group (< 10 surgeries) and the experienced group (≧ 10 surgeries).

### 2.3 Task and Evaluation

**FIGURE 2:**
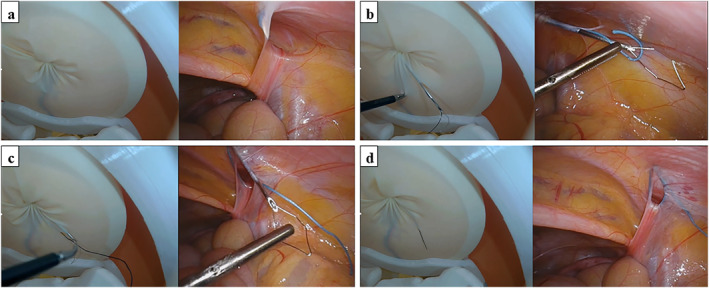
LPEC Surgery. (a) The needle device with a nonabsorbable suture material is inserted from outside the body into the preperitoneal space. (b) After half of the circuit suturing was completed, the needle is inserted into the abdominal cavity and the suture is released into the abdominal cavity. (c) After the needle without the suture material is advanced in the same manner for the opposite half of the circuit, the needle tip is inserted through the same hole into the abdominal cavity. (d) The needle device is removed from the abdomen together with the suture material.

The task was LPEC simulation surgeries using a disease‐specific simulator. In LPEC surgery, the orifice of the hernial sac is closed extraperitoneally with circuit suturing around the internal inguinal ring using a needle device. First, a needle device with nonabsorbable suture material was inserted from outside the body into the preperitoneal space (Figure 2a). The first half of circuit suturing began extraperitoneally from the anterior to the posterior edge of half of the internal inguinal ring. At this time, the needle was manipulated to cross the gonadal vessels and spermatic duct so as not to injure or bind them. After half of the circuit suturing was completed, the needle was inserted into the abdominal cavity and the suture was released into the abdominal cavity (Figure 2b). Once the needle without the suture material is returned to the puncture point in the preperitoneal space, the needle is extraperitoneally advanced in the same manner for the opposite half of the circuit, and the needle tip is inserted through the same hole into the abdominal cavity (Figure 2c). The suture material was held by a wire loop inside the needle. The needle device was then removed from the abdomen along with the suture material (Figure 2d). Circuit suturing was performed extracorporeally, and the internal inguinal ring was completely closed.

**FIGURE 3:**
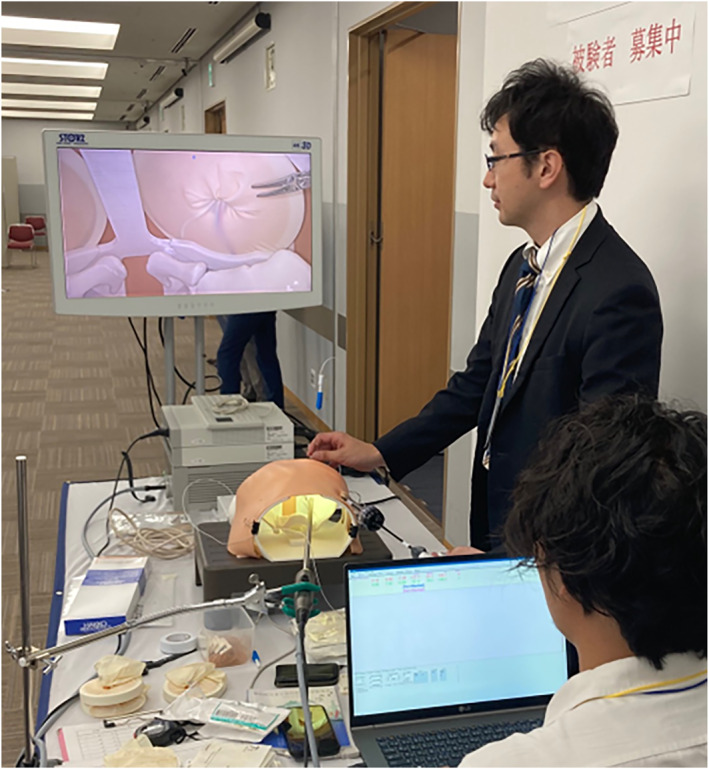
The setting of the simulation surgeries.

Participants stood on the right or left side of the simulator, whichever they preferred, and performed hernia surgery on the ipsilateral side. The port was inserted into the side of the abdomen on which the participants were standing, and a 5‐mm Kelly‐type forceps was used. A Lapa‐her‐closure (Hakko, Nagano, Japan) was used as the needle device for LPEC, and a 2‐0 silk blade suture was used for circuit suturing. A 30° 10‐mm 4k scope and 4 K 32‐inch monitor were used for laparoscopy in the simulated surgeries (Figure 3).

The task completion time, three‐dimensional characteristics of needle device manipulation, and the accuracy of needle device manipulation were evaluated as evaluation points of the LPEC simulation surgery. Regarding to three‐dimensional characteristics of needle device manipulation, the total path length, average velocity, and average acceleration of the needle device were evaluated by a magnetic three‐dimensional position‐measuring device (TrackSTAR; Northern Digital Inc., Waterloo, Canada). The magnetic three‐dimensional position‐measuring device's sensor was substituted by attaching it to the head of the needle for measurement, as attaching it to the tip of the needle would make the simulated surgery unfeasible. The accuracy of needle device manipulation was evaluated using a checklist with 2 points for each of the 7 assessment items, for a total of 14 points. The seven assessment items were as follows: needle insertion into the extraperitoneal space, number of mistake punctures of the peritoneum, needle insertion into the abdominal cavity, peritoneal skipping, ligation of extraperitoneal tissue, ligation of the spermatic duct, and ligation of gonadal vessels (Table 1).

**TABLE 1:** The checklist with two points for each of seven assessment items.

Assessment items	Assessment (points)
Needle insertion into the extraperitoneal space	Poor (0)・Fair (1)・Good (2)
Number of mistake punctures of the peritoneum	More than 1 time (0)・1 time (1)・0 times (2)
Needle insertion into the abdominal cavity	Skip (0)・Same hole or crossover (2)
Peritoneal skipping	More than 1/12 circle (0)・Less than 1/12 circle (1)・None (2)
Ligation of extraperitoneal tissue	More than 1/12 circle (0)・Less than 1/12 circle (1)・None (2)
Ligation of spermatic duct	Done (0)・None (2)
Ligation of gonadal vessels	Done (0)・None (2)

### 2.4 Questionnaire

After the simulation surgery, a questionnaire survey was administered to the experienced group. The questionnaire consisted of three questions: (1) Do you think the simulator reproduces the actual LPEC surgery? (2) Do you think the simulator is effective for novices learning needle manipulation?, (3) Do you think training using a simulator is effective for actual LPEC procedures?. The participants rated each questions on a 5‐point Likert‐type scale ranging from 1 (no) to 5 (yes).

### 2.5 Statistical Analyses

Data were analyzed using Microsoft Excel 2019 (Microsoft Corporation, Redmond, WA, USA). Quantitative results were analyzed using a univariate *t*‐test. Data are presented as means with standard deviations (SDs) and with significance set at *p* < 0.05.

### 2.6 Ethical Issues

The study design was approved by the Institutional Review Board of Kagoshima University (170361(635)‐2). All participants received an explanation of the study objective, and written informed consent was obtained from them during the study. The collected data were anonymized to maintain the privacy of the participants.

## 3 Results

### 3.1 Backgrounds of the Participants

Fifty‐three participants performed simulation surgery, of whom 35 were in the experienced group and 18 were in the novice group. The mean postgraduate experience was 3.9 years for the novice group and 12.4 years for the experienced group. More than 40 cases of LPEC procedures were performed by 80.0% of the experienced group (Table 2).

**TABLE 2:** Background of the participants.

	Novice group	Experienced group
Participants (*n*)	18	35
Postgraduate experience, years (mean)	3.9	12.4
Experiences of LPEC (*n* (%))		
0–9	18 (100.0)	—
10–19	—	1 (2.9)
20–29	—	5 (14.3)
30–39	—	1 (2.9)
40–49	—	5 (14.3)
50‐	—	23 (65.7)

### 3.2 Results of the LPEC Simulation Surgery

All 35 participants in the experienced group were able to complete the simulation surgery, whereas 16 (88.9%) in the novice group were able to complete it, which amounted to a significant difference (*p* = 0.013) (Table 3).

**TABLE 3:** Results of the LPEC simulation surgery.

	Novice group	Experienced group	*p*‐value
Completion of simulation surgery (*n* (%))	16 (88.9)	35 (100.0)	0.013
Task completion time (sec)	695.8 ± 255.0	308.1 ± 132.6	< 0.001
Three‐dimensional characteristics of needle device manipulation			
Total pass length (mm)	102678.1 ± 76999.3	37591.1 ± 3544.5	0.001
Velocity (mm/s)	136.8 ± 75.0	79.2 ± 40.1	0.002
Acceleration (mm/s^2^)	17775.5 ± 13128.5	8477.9 ± 8125.6	0.005
Accuracy of needle device manipulation evaluated using a checklist (max. 2 points per item)			
Needle insertion into the extraperitoneal space	2.0 ± 0.0	2.0 ± 0.0	—
Mistake puncture of peritoneum	0.8 ± 0.8	1.7 ± 0.5	< 0.001
Needle insertion into the abdominal cavity	1.6 ± 0.8	1.9 ± 0.3	0.046
Peritoneal skipping	1.4 ± 0.8	1.9 ± 0.3	0.008
Ligation of extraperitoneal tissue	1.4 ± 0.8	1.7 ± 0.5	0.143
Ligation of spermatic duct	2.0 ± 0.0	1.9 ± 0.3	0.162
Ligation of gonadal vessels	2.0 ± 0.0	2.0 ± 0.0	—
Total points (max. 14 points)	11.3 ± 2.4	13.1 ± 1.1	0.003

*Note:* Mean ± SD.

The experienced group had a significantly shorter task completion time than the novice group (308.1 ± 132.6 vs. 695.8 ± 255.0 s, *p* < 0.001). Furthermore, the experienced group had a significantly shorter total pass length of the needle device, slower average velocity of needle device, and lower average acceleration of needle device in comparison to the novice group (total pass length 37591.1 ± 3544.5 vs. 102678.1 ± 76999.3 mm, *p* = 0.001; average velocity 79.2 ± 40.1 vs. 136.8 ± 75.0 mm/s, *p* = 0.002, average acceleration 8477.9 ± 8125.6 vs. 17775.5 ± 13128.5 mm/s^2^, *p* = 0.005) (Table 3).

In the evaluation using a checklist, the experienced group had significantly fewer mistake punctures of peritoneum (1.7 ± 0.5 vs. 0.8 ± 0.8 points, *p* < 0.001), significantly more accurate needle insertion into the abdominal cavity (1.9 ± 0.3 vs. 1.6 ± 0.8 points, *p* = 0.046), and significantly fewer peritoneal skips (1.9 ± 0.3 vs. 1.4 ± 0.8 points, *p* = 0.008). The total points were also significantly higher in the experienced group (13.1 ± 2.4 vs. 11.3 ± 2.4 points, *p* = 0.003) (Table 3).

### 3.3 Results of the Questionnaire Survey

The response rate for the questionnaire survey was 100.0%. Participants in the experienced group evaluated the reproducibility of the actual surgery in the LPEC simulator. They also evaluated the usefulness of the LPEC simulator in helping novices learn to manipulate the needle device. Training in the LPEC simulator was highly effective for performing actual surgeries (Table 4).

**TABLE 4:** Results of the questionnaire survey.

	Response (mean ± SD)
Do you think the simulator reproduces the actual LPEC surgery well?	4.24 ± 0.85
Do you think the simulator is effective for novices to learn needle manipulation?	4.71 ± 0.61
Do you think training using the simulator is effective for actual LPEC procedures?	4.55 ± 0.78

*Note:* Responses are on a 5‐point Likert‐type scale ranging from 1 (no) to 5 (yes).

## 4 Discussion

We conducted a validation study of a disease‐specific simulator of the LPEC method for pediatric inguinal hernia that compared experienced pediatric surgeons and novices. This was one of the first studies to validate an LPEC simulator. The major findings of this study were as follows: (1) the experienced group had significantly better results in the simulation surgery than the novice group, which indicates that our simulator was able to successfully differentiate between experienced and novice surgeons and (2) the effectiveness of our simulator was very highly evaluated by experienced surgeons.

Instrumental motion tracking has been used as an objective assessment tool for surgical skills using a laparoscopic surgical simulator [3, 4, 5, 6]. Generally, the total path length decreases depending on the proficiency level [7, 8]. Improvements in the manipulation of forceps increase the speed and acceleration from a novice to an intermediate skill level; however, expert surgeons manipulate endoscopic forceps with slower speed, lower acceleration, and smoother movements than intermediate‐skilled surgeons, and higher acceleration is associated with jerkier forceps manipulation [8]. In the suturing task, slow manipulation and shorter path length increase the quality of the suturing procedure [9]. In the present study, the experienced group showed a significantly shorter total pass length, slower average velocity, and lower average acceleration of the needle device than the novice group, which implied leaner and smoother manipulation. These techniques lead to a shorter task completion time. The validity of a simulator can be established by its ability to differentiate between different skill levels [10]. Our simulator could clearly differentiate between novice and experienced surgeons, and the validity of the LPEC simulator was established.

In the evaluation of the accuracy of needle device manipulation using the checklist, the experienced group showed significantly better performance. Our checklist for the LPEC method could also clearly differentiate between novice and experienced surgeons. Better manipulation of the needle device, as measured by the three‐dimensional position‐measuring device, was also thought to have had a positive impact on the objective evaluation using the actual procedure checklist. Assessment and feedback of the surgeon's skill are considered very important in surgical training, and it has been found that educating novices with a checklist to assess skills in each step of the surgical procedure can significantly improve surgical outcomes [11, 12]. This checklist could be used in conjunction with the LPEC simulator to achieve higher training effectiveness in the education of novices.

The validity of the LPEC simulator was also proven using a questionnaire survey after simulation surgery. In the questionnaire survey, the evaluations of the participants of the experienced group indicated that the simulator reproduced the actual LPEC surgery well and was effective for novices to learn needle manipulation and that training using the simulator was effective for actual LPEC procedures. Eighty percent of the participants in the experienced group had performed > 40 LPEC procedures, and these high evaluations were considered reasonable.

As a limitation, this study only compared novice and experienced trainees, and it was not possible to verify whether novice trainees could actually train and acquire a better technique using the simulator. However, we succeeded in introducing LPEC to Nepal by simulation‐based training using this LPEC simulator [13]. Training using the LPEC simulator is considered an effective way to educate novices. In future studies, we aim to verify whether training on the simulator actually improves the novice technique and has a positive impact on actual surgical outcomes.

## 5 Conclusion

Our disease‐specific simulator for LPEC was able to clearly differentiate between novice and experienced surgeons, and the effectiveness of our simulator was highly evaluated by experienced surgeons.

## Author Contributions

**Masakazu Murakami:** conceptualization, data curation, formal analysis, funding acquisition, investigation, methodology, project administration, resources, software, validation, visualization, writing – original draft, writing – review and editing. **Yumiko Tabata:** investigation. **Yumiko Iwamoto:** investigation. **Masato Ogata:** investigation. **Lynne Takada:** investigation. **Chihiro Kedoin:** investigation. **Yudai Tsuruno:** investigation. **Koshiro Sugita:** investigation. **Keisuke Yano:** investigation. **Shun Onishi:** investigation. **Takafumi Kawano:** investigation. **Satoshi Ieiri:** supervision, writing – review and editing.

## Conflicts of Interest

The authors declare no conflicts of interest.
